# Exploring the Future of Polyhydroxyalkanoate Composites with Organic Fillers: A Review of Challenges and Opportunities

**DOI:** 10.3390/polym16131768

**Published:** 2024-06-22

**Authors:** Abhishek Thakur, Marta Musioł, Khadar Duale, Marek Kowalczuk

**Affiliations:** Centre of Polymer and Carbon Materials, Polish Academy of Sciences, M. Curie-Skłodowskiej 34, 41-800 Zabrze, Poland; mmusiol@cmpw-pan.pl (M.M.); kduale@cmpw-pan.pl (K.D.)

**Keywords:** Polyhydroxyalkanoates (PHAs), polymer composites, biocomposites, waste natural fillers

## Abstract

Biopolymers from renewable materials are promising alternatives to the traditional petroleum-based plastics used today, although they face limitations in terms of performance and processability. Natural fillers have been identified as a strategic route to create sustainable composites, and natural fillers in the form of waste by-products have received particular attention. Consequently, the primary focus of this article is to offer a broad overview of recent breakthroughs in environmentally friendly Polhydroxyalkanoate (PHA) polymers and their composites. PHAs are aliphatic polyesters obtained by bacterial fermentation of sugars and fatty acids and are considered to play a key role in addressing sustainability challenges to replace traditional plastics in various industrial sectors. Moreover, the article examines the potential of biodegradable polymers and polymer composites, with a specific emphasis on natural composite materials, current trends, and future market prospects. Increased environmental concerns are driving discussions on the importance of integrating biodegradable materials with natural fillers in our daily use, emphasizing the need for clear frameworks and economic incentives to support the use of these materials. Finally, it highlights the indispensable need for ongoing research and development efforts to address environmental challenges in the polymer sector, reflecting a growing interest in sustainable materials across all industries.

## 1. Introduction

Since the introduction of different petrochemical-based polymer manufacturing processes, the plastic industry has grown significantly. Plastics have several advantages, including durability, lightweight, and affordability, with approximately half of all plastic materials utilized for single use in everything from packaging to infrastructure components. More than 400 million tons of plastics are produced worldwide every year, with little recycling and significant environmental damage, requiring urgent action. The projected increase in primary plastic waste output by 2050, reaching 25 billion metric tons, has prompted a significant movement toward sustainability. By 2025, leading plastic packaging companies intend to include 100% recycled, biodegradable, or reusable plastics in their goods [1]. The EU and the High Ambition Coalition are leading the discussions for a legally enforceable instrument to reduce plastic pollution, which is a critical first step toward long-term solutions [2]. Plastic’s environmental lifespan is still unknown due to its recent mass manufacture, with most varieties needing thousands of years to decompose depending on local environmental variables [3]. As a result, solid waste has become a major global problem, leading to a shift towards environmentally friendly materials made from natural fillers and polymers to mitigate this problem. Polymers are made up of long, repetitive chains of molecules with varying bonding and structural properties. Polymer composites are created by adding polymers to other materials, nanomaterials, additives, and other compounds that make them better and cheaper than traditional materials such as metal, wood, and leather [4]. The aim is to obtain a material with improved properties such as increased strength, durability, increased toughness, lower density, and lightweight design for a range of engineering applications [5]. In addition to being biodegradable, biocomposites comprised of biological components also have the advantage of being non-toxic and safer to work with, offering advantages including decreased pollutant emissions and less dependence on non-renewable resources [6]. 

**Figure 1:**
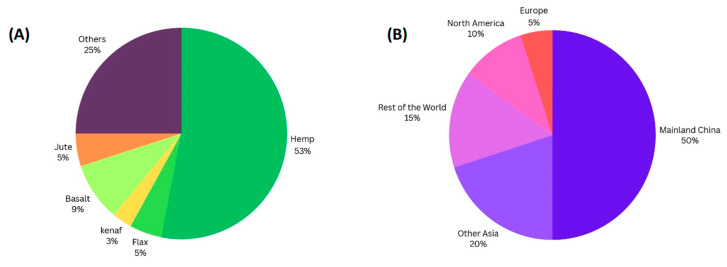
(**A**) Market use of natural fibers in percentage [12]; (**B**) world consumption of natural and man-made fibers by region [13].

Polyhydroxyalkanoates (PHAs) are microbial polyesters produced by several bacteria under nutritional stress circumstances that have thermoplastic characteristics similar to conventional polymers [7]. However, their relatively high cost limits their employment in single-use goods, mostly restricted to high-value applications such as the medical and pharmaceutical industries [8]. Increasing the use of PHA composites requires a reduction in the production costs of virgin PHAs and the use of low-cost natural and inorganic fillers. For instance, composite materials, which contain lignocellulosic fibers and natural fillers from agricultural and industrial crops such as maize, wheat, bagasse, and others, have been shown to have advantages such as low density and valuable mechanical properties and are used in packaging, automotive, and construction industries [9]. Fiber-based composites, whether synthetic or plant-derived, are termed bio-based if they utilize biodegradable polymers offering benefits like enhanced toughness and lower cost. Plant fibers like pineapple, coir, and jute are increasingly favored over synthetic materials for their biodegradability, renewability, and higher resistance in load-bearing applications [10]. Although natural and synthetic biodegradable polymers are highly publicized, they often do not meet the technical criteria and their high cost hinders their wide use. However, upgrades through chemical or physical changes, reactive processing, and polymer blends can overcome these limitations and improve performance in a variety of applications [11]. The market use of natural fibers has also increased in recent times. Companies are looking for alternatives that can replace their current products and lead the world toward a sustainable and eco-friendly environment. Environmentally friendly biocomposites, which use natural fillers such as hemp or flax for biodegradability, lower density, and cost-effectiveness, have attracted considerable interest [12]. Natural fiber composites (NFCs) like wood, flax, hemp, kenaf, coir, sisal, and others serve diverse applications. Wood fibers are prized for construction and furniture, while flax offers lightweight strength for automotive and packaging. Hemp gains popularity in automotive and textiles for sustainability. Kenaf suits automotive interiors and construction. Coir aids erosion control and interiors. Sisal excels in industrial uses. Other fibers like jute, abaca, and banana find varied applications, contributing to sustainability across sectors [13]. Figure 1A below illustrates the use of natural fibers as a percentage (based on [14]) and Figure 1B shows the consumption of natural and man-made fibers by region (based on [15]).

## 2. PHA Composites

PHAs are biodegradable polymers generated by microorganisms under nutrient-limiting conditions and provide a sustainable alternative to traditional petroleum-based plastics [16]. PHAs, thermoplastic materials similar to petrochemical plastics, are produced from hydroxyalkanoic acids. The properties of a monomer are determined by the length of its carbon chain; medium-chain PHAs have elastomeric properties, while short-chain PHAs are similar to conventional polymers such as polypropylene [17]. Common PHA polymers such as PHB and PHBV act as substrates for bacterial energy storage, just as plant starch and animal fats do. Energy-rich feedstocks are converted into fatty acids throughout the industrial PHA manufacturing process. Purified polymer powders or particles are produced by the separation, dissolution, and extraction cycles that cells go through [18]. Poly-3-hydroxybutyrate (P3HB or PHB) is probably the most common and widely studied polyhydroxyalkanoate, derived from the fermentation of sugars or fats by bacteria and characterized by its high crystallinity and brittleness.

Biocomposites, where natural fibers from renewable by-products are blended with PHA, are driving the development of sustainable materials. Biocomposites, which blend natural fibers from renewable by-products with PHAs, are a driving force for sustainable material innovation. Natural fibers from sources such as wood and agricultural waste are ideal to use as reinforcement materials in composites due to their favorable properties such as high strength, low density, and renewability [19]. Biocomposites, which combine natural fibers and PHAs, are used in a variety of packaging and automotive applications [20]. They improve mechanical properties, accelerate biodegradation, and reduce environmental impact [21]. These biocomposites are promising as long-term replacements. Researchers are also investigating the production of PHAs in genetically modified bacterial strains [22].

**Figure 2:**
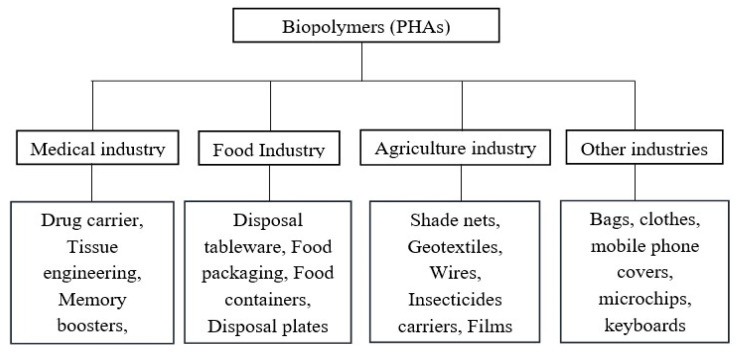
Biopolymer (PHAs) usage in different industries [22].

The increasing use of PHA-based materials in mainstream applications can be attributed to their biodegradability and biocompatibility. Co-polymerization with copolymers, e.g., poly (ethylene glycol) (PEG), can modify the mechanical and thermal characteristics of PHA [23]. The mechanical properties of biocomposites containing fibers such as hemp, abaca, flax, and kenaf surpass those of pure polymers. The properties of PHA composites are influenced by factors such as fiber modulus, aspect ratio, morphology, and interfacial adhesion. Efforts to improve PHA biodegradation focus on natural fillers and fibers, which introduce channels for water and enzymes into the polymer matrix, accelerating degradation [24]. PHAs are useful biomaterials that, due to their structural variability and adaptable properties, hold great promise for a wide range of applications in many sectors. Despite the aforementioned obstacles, such as high production costs and molecular instability, there is a constant research effort to decrease costs and broaden PHA’s commercial interest, making it a versatile material with promising applications. PHAs are now embedded in many parts of our daily lives and used in all industries, as illustrated in Figure 2 [25].

Global manufacturing constraints limit the development of PHA-based goods. In recent years, the growth rate of manufacturing capacity for bio-based polymers has declined, from 10% per year between 2012 and 2014 to 4% per year between 2014 and 2016, which is in line with the growth rate of global polymer production as a whole. Although PHAs have the largest growth rate, bio-based polymers as a whole are expanding at a slower rate than expected [26]. Low oil prices, unfavorable policy environments in most countries, a slower-than-expected rate of development in terms of capacity utilization, and social reservations about embracing novel bio-products are some of the reasons behind this [27]. 

Mathematical models can greatly assist both the design and operation of the composite. The development of artificial intelligence (AI) and machine learning (ML) techniques in particular have become increasingly important in recent years and there is no field in which they have not made their mark. Recent developments in this field show that AI is causing a composite revolution by offering unprecedented opportunities for material modeling and design [28,29]. AI can examine large datasets and find new composite formulations with exact properties by using techniques like machine learning and deep learning [30]. This improves durability and performance through precise material behavior prediction, manufacturing process optimization, and design of new structures, and also speeds up simulation time and thus material discovery. Automation of material characterization expedites research, and multi-scale modeling offers a thorough comprehension of intricate ideas. AI-powered sustainability programs enhance formulations even more, cutting down on waste and resource use. In a recent study, Luna and co-workers demonstrated a hybrid model for the operation of a bioreactor containing the PHA-producing P. putida strain GPo1 [31]. The model uses a neural network to take into account growth, uptake, and production rates, where the authors used Monod-type kinetic expressions to support the calculation of these quantities, as they cannot be measured directly. In another example, ML was used to rapidly predict the properties of multicomponent PHA-based copolymers to demonstrate the powerful structure-property mapping capabilities of ML-based techniques in PHA studies [32].

## 3. Biodegradable Polymer Composites with Natural Waste Fillers

With increasing environmental concerns, countries have made significant investments in renewable resources across a range of industries, and these materials include wood flour waste, natural fibers, and agricultural by-products such as corn cobs, rice husks, and rice straw [33]. However, the efficient use of waste substrates has always been a major challenge. While some substrates are now used to some extent, millions of tons of waste remain unused, providing opportunities for a variety of everyday applications. The waste with its volume and applications is illustrated below in Table 1 [34].

Biodegradable polymer research highlights the under-exploited potential of organic waste by turning to renewable resources and developing biodegradable aliphatic polymers. Additionally, research has demonstrated that it is possible to use organic waste to create valuable biomaterials, providing an opportunity to replace current waste management technologies with more environmentally friendly ones [45]. In a study of biochar composites, it has been investigated that it could be used to make new materials. The mixing of biochar with Poly(1,4-butylene adipate-co-1,4-butylene terephthalate) PBAT/Polylactide (PLA) reduces the surface resistivity. It showed increased elastic modulus with filler content, while thermal stability was minimally affected. Overall, biochar incorporation shows promise for specialized applications as an antistatic agent [46]. The application of biopolymers and alternative manufacturing methods presents several opportunities. Given the use of organic waste, it can be used as fermented sugars. Many different kinds of organic waste that can be used as sustainable feedstocks for biopolymer synthesis are agricultural residues, garden waste, forest waste, livestock waste, paper waste, wastewater sludge, municipal solid waste, and kitchen garbage [47]. The cost and availability of raw materials in different parts of the world have a major impact on biopolymer production processes. Composite materials are being investigated as potential replacements for conventional materials by scientists and researchers who are constantly looking for materials with high strength and low density. Composite materials, which consist of two or more insoluble components, have special properties that distinguish them from their constituent parts [48]. In particular, plant fiber-reinforced polymer composites are becoming more popular because of their prospective applications and biodegradability. With natural composites, which can be fully or partially biodegradable depending on the mix of biobased fibers and polymers, the emphasis is on sustainability. Because they are lightweight and environment friendly, these materials are becoming more and more in demand in the automotive and construction sector [49]. Plant fibers with varying mechanical and tribological capabilities include abaca, sisal, jute, rice husk, and kenaf. Sisal and jute have better flexural properties [50]. A combination of fiber volume, orientation, and surface modification enables hybrid plant fiber-reinforced composites to perform better than glass fiber laminates. These composites are excellent in mechanical, acoustic, and thermal qualities, making them useful for a variety of uses [51]. Many waste products have been considered for PHA production, including vegetable oils, molasses, animal fats high in carbon and nitrogen, and agricultural leftovers. Bacteria like Escherichia coli, Alcaligenes latus, and Pseudomonas species manufacture PHA from these substrates; yields are optimized by the use of methodologies like Design of Experiments (DOEs) [52]. PHA synthesis has increased due to developments in fermentation techniques and the availability of cheap waste substrates, creating waste management solutions that are both economical and advantageous for the environment [53]. Taking advantage of biorefineries for sustainability is another idea, which involves using organic resources like food waste and specialty crops to turn biomass into marketable goods. Though cost-effective ways to use fermentable sugars from lignocellulose are still lacking, integrating PHA production within biorefineries employing waste streams like glycerol and lignocellulosic sugars shows promise [54]. Biorefinery becomes sustainable when organic waste acceptance is conditioned by charging waste management costs. Long-term survival requires overcoming logistical, technological, financial, moral, and legal challenges, especially given the pressing need to address waste issues and transition to a circular economy [55]. Engineered materials utilize polymer composites, which are composed of two or more insoluble components, combining a thermoplastic or thermoset matrix with organic or inorganic fillers like wood flour or fibers, to achieve desired results [56]. Although natural fibers are mechanically strong and biodegradable, their hydrophilic nature can make them incompatible with conventional plastic materials [57]. Component composition, ambient factors, and surface properties all affect how quickly composite materials biodegrade; natural fillers may encourage microbial activity and accelerate breakdown [58].

## 4. Applications and Properties

**Figure 3:**
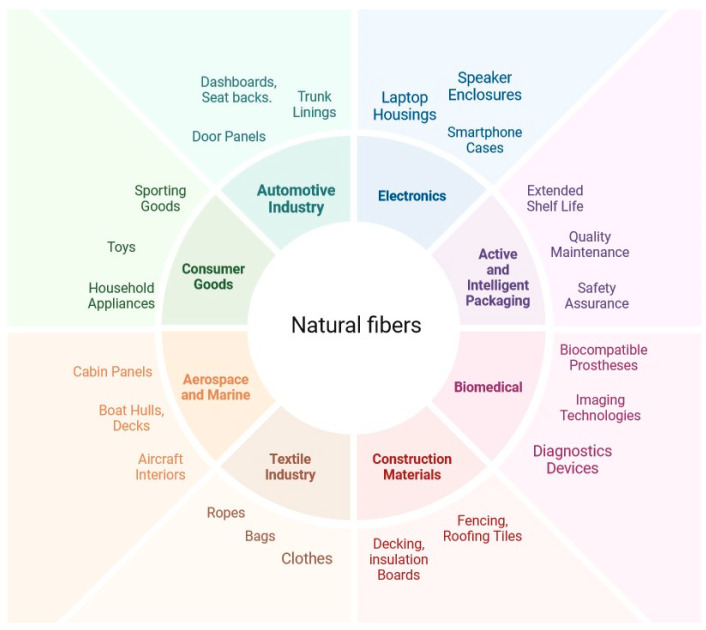
Natural fiber usage with respective industries.

Natural fibers and PHAs have numerous applications that promote environmental preservation and sustainable development. Natural fibers, derived from plants or animals, are biodegradable, lightweight, and renewable, and have many day-to-day applications [42] that are illustrated below in Figure 3.

PHAs are utilized in waste management, biomedical engineering, and packaging because of their reputation for biodegradability and biocompatibility [59]. PHAs have a number of advantages, including being biodegradable, non-toxic, non-immunogenic, non-carcinogenic, and non-thrombogenic. They are created from renewable resources, which assist in limiting pollution, increasing agricultural production, mitigating global warming, and promoting healthy aquatic habitats. Domestic plastics, biomedical devices, pharmaceuticals, adhesives, cosmetics, the oil sector, textiles, and automobiles are all examples of applications [60]. Similarly, natural fibers have different qualities depending on things like crop location, processing techniques, and chemical composition. Recent years have seen a surge in interest in natural fiber reinforcement in composite materials because of its exceptional mechanical properties. The complete biodegradability, renewable nature, eco-friendliness, affordability, availability, and low density of these fibers are widely recognized [61]. Because plant fibers are biological and strongly biodegradable, they contribute to ecological balance while being heavier than mineral fibers. Natural fiber-reinforced polymers are widely utilized in industry due to their great performance when their life cycle comes to a conclusion; the CO_2_ they absorb during growing balances the CO_2_ they release while burning. Furthermore, the recycling of composite materials is made easier by the low friction potential of plant fibers [62]. Natural fibers are more lightweight than fibers made of minerals, but they nevertheless perform well and support ecological balance. Natural fibers, despite potentially having lower mechanical properties compared to glass or mineral-based fibers, offer similar stiffness and tensile strength, making them valuable reinforcements in composite applications [63]. New applications for bio-based composites and nanocomposites have been developed through the use of green-generated nanoparticles and by-products from the agricultural and food industries. Moreover, with applications ranging from paint removal to water desalination, research on bio-based composites and nanocomposites for water purification and pollution recovery is crucial for the sustainable use of clean water [64]. They also have potential in rigid electronics components and energy storage systems, as demonstrated by biocomposite membranes that include microcrystalline cellulose in polymer matrices [65]. Research on wood panels made from industrial residues and environmentally friendly adhesives as substitutes for synthetic counterparts also shows that bio-based materials have the potential to be used in the construction industry. Polyester systems are promising as paper coatings for multi-layer packaging materials [66], while jute fibers, which have been observed for their biodegradability and compatibility with biodegradable polymers, are used in environmentally friendly packaging solutions [67]. 

## 5. Current Developments

In the global market, there has been a noticeable increase in demand for natural and bio-based fillers. The use of fillers increased in the USA in the last decades, from 525,000 tons to 1,925,000 tons [6]. Adding natural fillers to biodegradable polymer matrices increases the versatility and application possibilities of eco-friendly products. Economic considerations are crucial for manufacturers, but the possibility of changing product properties by varying the amount of filler opens up new applications [68]. Through careful selection of polymer matrix and filler types, content ratios, and size distribution, manufacturers can customize the final properties of these materials to suit a variety of applications. For example, the addition of cork as a natural filler in P(3HB-co-4HB) composites has shown improved thermal stability, resulting in slower degradation rates compared to the pure matrix in different environments [69]. In other findings, composites comprising P(3HB-co-4HB) and wood flour exhibited accelerated degradation processes under both laboratory and industrial composting conditions; the addition of wood flour significantly changes the thermal and mechanical properties of the composites. The presence of acidic by-products from hydrolytic degradation and compounds derived from the filler contributes to enhanced thermal stability. This gives great promise in food packaging applications [70]. 

Research on biomass-based polymeric substitutes and composites has been driven by concerns about the depletion of fossil fuels and their potential environmental effects on composites [71]. This change is being reflected in the sharp increase in research publications on biocomposites that emphasize the ingenuity and potential of these materials. The key objective is to shift towards a circular or sustainable economy that focuses on the use of bio-based and biodegradable products. Reusability, recyclability, and biodegradability have been prioritized in the optimization of the life cycle through recent programs. However, creating fully sustainable goods and procedures calls for upgrades to production techniques, waste management systems, and retail distribution networks [72]. This has led to a rise in interest in fully integrated production systems, such as biorefineries, which integrate several manufacturing phases in an effort to minimize emissions, transportation expenses, and waste formation. To realize the full potential of bioplastics and biocomposites in advancing global sustainability, however, comprehensive and reliable information about their sustainability is necessary [73]. For these resources to be sustainable, equitable farming systems that respect both the environment and society must be developed. To achieve this, the methods of producing biomass must be sure that they promote the welfare of farmers, communities, and the environment. However, while biopolymers have inherent advantages for environmentally friendly uses such as PHA, cost remains an issue [74]. PHAs, or naturally occurring polyesters produced by bacteria under harsh conditions during the fermentation of fats or sugars, have enormous potential for the development of sustainable materials. Reusing a mass of residues can save costs and increase sustainability by using biomass from food production by-products and waste from the agricultural and food industries [75]. When natural fillers are added to biopolymers to enhance their properties, green composites are produced. For instance, rice husk flour (RHF), compatibilizers, and initiators have been combined with commercial poly(3-hydroxybutyrate) (PHB) and poly(3-hydroxybutyrate-co-3-hydroxyvalerate) (PHBV) made from fruit pulp waste to successfully construct green composites [76]. Using this unique approach, films with better thermal stability, balanced strength and ductility, and robust barrier properties have been created, making them suitable for rigid packaging applications. Partnerships between researchers, producers, and other stakeholders demonstrate a commitment to developing environmentally friendly and economically successful sustainable materials [77]. The increasing demand for these fillers reflects the dynamic developments in material design, where solutions driven by specific application requirements are prioritized.

## 6. Challenges and Future Aspects

Commercializing PHAs offers significant obstacles due to their higher production costs as compared to petroleum-based polymers, as well as market acceptance concerns becoming worse due to competition from established brands. Despite their competitiveness in specific applications, PHAs face price parity difficulties with petroleum-based polymers [78]. For instance, utilizing lignocellulosic waste presents challenges due to incompatibilities between the hydrophilic nature of natural fillers and the hydrophobicity of the thermoplastic matrix, affecting mechanical properties dependent on interfacial adhesion [79]. While water-based polymer composites have some advantages, they also face issues such as lower mechanical qualities, difficulties in optimizing filler–matrix interaction, restricted recyclability, and high energy requirements at the end of life. However, green synthesis methods and the use of recycled or biodegradable polymers show promise for improving waste management practices, exploring new markets, and tackling issues in polymer composites [80].

Future research aims to optimize PHA production, reduce costs, enhance stability, and explore high-value applications, leveraging their diverse structures and adaptive features [81]. The creative use of waste materials, focus on improving material compositions and processing techniques, and development of hybrid materials for the construction (for example, the use of bamboo in South Asia for different constructions [82]) and automotive industries show promise, along with the creation of affordable and biodegradable resins and adhesives using waste-derived resources [83]. Achieving superior mechanical characteristics and filler–matrix interactions through chemical changes is the main goal of research on biofiber-reinforced polymer composites. Developments in waste-based polymer composites are being driven by continuous efforts in ecologically friendly synthesis techniques and the use of waste-derived components [84]. Variability in waste composition, limited bacterial strains, metabolic engineering, and PHA extraction are some challenges that require ongoing research and development to realize the full potential of PHAs as sustainable biopolymers [85]. To promote widespread usage of sustainable materials across industries, future efforts should concentrate on optimization, technological advancements, and environmentally responsible solutions [86]. Research on biodegradable microplastics (BMPs) highlights their complex environmental interaction and potential ecological and health impacts. BMPs, needing specific conditions to degrade, can accumulate debris and foster biofilm formation, hindering decomposition. Acting as vectors for contaminants and microbes, BMPs’ persistence and long-term effects are under investigation, with concerns over their negative ecological impact on aquatic ecosystems and food webs. Understanding BMPs’ role in environmental contamination is crucial, requiring standardized toxicity testing and regulatory measures [87]. Concerns about the biodegradation of widely used biodegradable polymers like PLA and PBAT have led to the production of persistent Bio MPs, raising questions about their environmental impact and potential threats to food security. Studies suggest that Bio MPs may have heightened toxicity compared to nondegradable microplastics, with significant gaps in knowledge remaining regarding their quantities, toxicological effects, and interactions with other pollutants [88]. 

## 7. Conclusions

Switching to sustainable materials, notably, the usage of PHAs in conjunction with natural fillers and fibers, is critical in resolving the environmental challenges caused by traditional plastics. PHAs have major advantages such as biodegradability and biocompatibility, making them appropriate for a wide range of applications, including packaging, automotive, and biomedical areas. However, the high production costs and technological constraints of PHAs provide obstacles that must be solved. The addition of natural fillers derived from agricultural and industrial waste not only improves the mechanical characteristics and biodegradability of PHA composites, but also provides an environmentally responsible alternative that minimizes reliance on nonrenewable resources. 

Advances in artificial intelligence (AI) and machine learning (ML) are critical in optimizing the design and manufacturing processes of these composites, allowing the identification of new formulations with desired qualities. Despite these advances, concerns such as economic feasibility, filler–matrix compatibility, and the need for improved waste management systems remain. Future research should focus on lowering PHA production costs, increasing stability, and broadening their applications. Using biorefineries, enhancing filler–matrix interactions, and generating hybrid materials are all essential techniques. Addressing the environmental impact of biodegradable microplastics (BMPs) by regulatory measures and uniform testing is also critical. In conclusion, PHA-based biocomposites with natural fillers have considerable potential for sustainable materials, and ongoing research and technological improvements are crucial to overcoming existing hurdles and reaching a circular economy. 

## Figures and Tables

**Table 1:** Application of waste with estimated volume.

Waste Substrate	Commercial Volume (Million Tons/Year)	Applications	References
Food waste	931	Bio-gas	[35]
Animal waste	1400	Fertilizers, bio-energy	[36]
Agriculture waste	2500	Recycling materials	[37]
Municipal waste	2240	Energy resource	[38]
Industrial waste	84.5	Coal Combustion products	[39]
Sewage waste	10.4	Fertilizer, soil improver	[40]
Forest residue	4600	Bio-fuel production	[41]
Paper waste	67.4	Newspapers, towels	[42]
Wastewater	0.264–0.600	Treated wastewater	[43]
Lignocellulosic waste	2009	Animal feed	[44]

## References

[1] Tumu K., Vorst K., Curtzwiler G. (2023). Global Plastic Waste Recycling and Extended Producer Responsibility Laws. J. Environ. Manag..

[2] Xu Q., Zhang M., Han S. (2024). Reflections on the European Union’s Participation in Negotiations of the Global Plastic Pollution Instrument under International Environmental Law. Front. Mar. Sci..

[3] Andrady A.L. (1994). Assessment of Environmental Biodegradation of Synthetic Polymers. J. Macromol. Sci. Part C Polym. Rev..

[4] Saleh Alghamdi S., John S., Roy Choudhury N., Dutta N.K. (2021). Additive Manufacturing of Polymer Materials: Progress, Promise and Challenges. Polymers.

[5] Bhong M., Khan T.K., Devade K., Krishna B.V., Sura S., Eftikhaar H.K., Thethi H.P., Gupta N. (2023). Review of Composite Materials and Applications. Mater. Today Proc..

[6] Zaaba N.F., Ismail H. (2019). Thermoplastic/Natural Filler Composites: A Short Review. J. Phys. Sci..

[7] Philip S., Keshavarz T., Roy I. (2007). Polyhydroxyalkanoates: Biodegradable Polymers with a Range of Applications. J. Chem. Technol. Biotechnol. Int. Res. Process Environ. Clean Technol..

[8] Mukherjee A., Koller M. (2023). Microbial polyHydroxyAlkanoate (PHA) Biopolymers—Intrinsically Natural. Bioengineering.

[9] Cinelli P., Mallegni N., Gigante V., Montanari A., Seggiani M., Coltelli M.B., Bronco S., Lazzeri A. (2019). Biocomposites Based on Polyhydroxyalkanoates and Natural Fibres from Renewable Byproducts. Appl. Food Biotechnol..

[10] Mohit H., Mavinkere Rangappa S., Siengchin S., Gorbatyuk S., Manimaran P., Alka Kumari C., Khan A., Doddamani M. (2022). A Comprehensive Review on Performance and Machinability of Plant Fiber Polymer Composites. Polym. Compos..

[11] Dacko P., Kowalczuk M., Janeczek H., Sobota M. (2006). Physical Properties of the Biodegradable Polymer Compositions Containing Natural Polyesters and Their Synthetic Analogues. Macromolecular Symposia.

[12] Das P.P., Chaudhary V. (2021). Moving towards the Era of Bio Fibre Based Polymer Composites. Clean. Eng. Technol..

[13] Thapliyal D., Verma S., Sen P., Kumar R., Thakur A., Tiwari A.K., Singh D., Verros G.D., Arya R.K. (2023). Natural Fibers Composites: Origin, Importance, Consumption Pattern, and Challenges. J. Compos. Sci..

[14] Ramu S., Senthilkumar N. (2022). Approaches of Material Selection, Alignment and Methods of Fabrication for Natural Fiber Polymer Composites: A Review. J. Appl. Nat. Sci..

[15] Townsend T., Kozłowski R.M., Mackiewicz-Talarczyk M. (2020). 1B—World Natural Fibre Production and Employment. Handbook of Natural Fibres.

[16] Akinmulewo A.B., Nwinyi O.C. (2019). Polyhydroxyalkanoate: A Biodegradable Polymer (a Mini Review). J. Phys. Conf. Ser..

[17] Vannini M., Marchese P., Sisti L., Saccani A., Mu T., Sun H., Celli A. (2021). Integrated Efforts for the Valorization of Sweet Potato By-Products within a Circular Economy Concept: Biocomposites for Packaging Applications Close the Loop. Polymers.

[18] Sun J., Shen J., Chen S., Cooper M.A., Fu H., Wu D., Yang Z. (2018). Nanofiller Reinforced Biodegradable PLA/PHA Composites: Current Status and Future Trends. Polymers.

[19] Thyavihalli Girijappa Y.G., Mavinkere Rangappa S., Parameswaranpillai J., Siengchin S. (2019). Natural Fibers as Sustainable and Renewable Resource for Development of Eco-Friendly Composites: A Comprehensive Review. Front. Mater..

[20] Poltronieri P., Kumar P. (2017). Polyhydroxyalkanoates (PHAs) in Industrial Applications. Handb. Ecomater..

[21] Raj H., Tripathi S., Bauri S., Choudhary A., Mandal S., Maiti P. (2023). Green Composites Using Naturally Occurring Fibers: A Comprehensive Review. Sustain. Polym. Energy.

[22] Dobrogojski J., Spychalski M., Luciński R., Borek S. (2018). Transgenic Plants as a Source of Polyhydroxyalkanoates. Acta Physiol. Plant..

[23] Chodak I. (2008). Polyhydroxyalkanoates: Origin, Properties and Applications. Monomers, Polymers and Composites from Renewable Resources.

[24] Maiti S., Islam M.R., Uddin M.A., Afroj S., Eichhorn S.J., Karim N. (2022). Sustainable Fiber-reinforced Composites: A Review. Adv. Sustain. Syst..

[25] Abd El-malek F., Khairy H., Farag A., Omar S. (2020). The Sustainability of Microbial Bioplastics, Production and Applications. Int. J. Biol. Macromol..

[26] Aeschelmann F., Carus M. (2015). Bio-Based Building Blocks and Polymers in the World. Ind. Biotechnol..

[27] Vandi L.-J., Chan C.M., Werker A., Richardson D., Laycock B., Pratt S. (2018). Wood-PHA Composites: Mapping Opportunities. Polymers.

[28] Liu X., Tian S., Tao F., Yu W. (2021). A Review of Artificial Neural Networks in the Constitutive Modeling of Composite Materials. Compos. Part B Eng..

[29] Fang J., Xie M., He X., Zhang J., Hu J., Chen Y., Yang Y., Jin Q. (2022). Machine Learning Accelerates the Materials Discovery. Mater. Today Commun..

[30] Kibrete F., Trzepieciński T., Gebremedhen H.S., Woldemichael D.E. (2023). Artificial Intelligence in Predicting Mechanical Properties of Composite Materials. J. Compos. Sci..

[31] Luna M.F., Ochsner A.M., Amstutz V., von Blarer D., Sokolov M., Arosio P., Zinn M. (2021). Modeling of Continuous PHA Production by a Hybrid Approach Based on First Principles and Machine Learning. Processes.

[32] Bejagam K.K., Lalonde J., Iverson C.N., Marrone B.L., Pilania G. (2022). Machine Learning for Melting Temperature Predictions and Design in Polyhydroxyalkanoate-Based Biopolymers. J. Phys. Chem. B.

[33] Kalak T. (2023). Potential Use of Industrial Biomass Waste as a Sustainable Energy Source in the Future. Energies.

[34] Jaffur N., Kumar G., Jeetah P., Ramakrishna S., Bhatia S.K. (2023). Current Advances and Emerging Trends in Sustainable Polyhydroxyalkanoate Modification from Organic Waste Streams for Material Applications. Int. J. Biol. Macromol..

[35] Said Z., Sharma P., Nhuong Q.T.B., Bora B.J., Lichtfouse E., Khalid H.M., Luque R., Nguyen X.P., Hoang A.T. (2023). Intelligent Approaches for Sustainable Management and Valorisation of Food Waste. Bioresour. Technol..

[36] Pagliari P., Wilson M., He Z. (2020). Animal Manure Production and Utilization: Impact of Modern Concentrated Animal Feeding Operations. Anim. Manure: Prod. Charact. Environ. Concerns Manag..

[37] Ogbu C.C., Okey S.N. (2023). Agro-Industrial Waste Management: The Circular and Bioeconomic Perspective. Agricultural Waste-New Insights.

[38] He D., Hu H., Jiao F., Zuo W., Liu C., Xie H., Dong L., Wang X. (2023). Thermal Separation of Heavy Metals from Municipal Solid Waste Incineration Fly Ash: A Review. Chem. Eng. J..

[39] Abdellatief M., Al-Tam S.M., Elemam W.E., Alanazi H., Elgendy G.M., Tahwia A.M. (2023). Development of Ultra-High-Performance Concrete with Low Environmental Impact Integrated with Metakaolin and Industrial Wastes. Case Stud. Constr. Mater..

[40] Bagheri M., Bauer T., Burgman L.E., Wetterlund E. (2023). Fifty Years of Sewage Sludge Management Research: Mapping Researchers’ Motivations and Concerns. J. Environ. Manag..

[41] Tripathi N., Hills C.D., Singh R.S., Atkinson C.J. (2019). Biomass Waste Utilisation in Low-Carbon Products: Harnessing a Major Potential Resource. NPJ Clim. Atmos. Sci..

[42] EPA (2020). National Overview: Facts and Figures on Materials, Wastes and Recycling. Retrieved Novemb..

[43] Mavugara R., Matsa M.M. (2024). Resource Recovery from Municipal Wastewater Treatment Plants: The Zimbabwean Perspective. Circ. Econ. Sustain..

[44] Singh N., Singhania R.R., Nigam P.S., Dong C.-D., Patel A.K., Puri M. (2022). Global Status of Lignocellulosic Biorefinery: Challenges and Perspectives. Bioresour. Technol..

[45] Acharjee S.A., Bharali P., Gogoi B., Sorhie V., Walling B., Alemtoshi (2023). PHA-Based Bioplastic: A Potential Alternative to Address Microplastic Pollution. Water Air Soil Pollut..

[46] Musioł M., Rydz J., Janeczek H., Kordyka A., Andrzejewski J., Sterzyński T., Jurczyk S., Cristea M., Musioł K., Kampik M. (2022). (Bio) Degradable Biochar Composites–Studies on Degradation and Electrostatic Properties. Mater. Sci. Eng. B.

[47] Battista F., Frison N., Pavan P., Cavinato C., Gottardo M., Fatone F., Eusebi A.L., Majone M., Zeppilli M., Valentino F. (2020). Food Wastes and Sewage Sludge as Feedstock for an Urban Biorefinery Producing Biofuels and Added-value Bioproducts. J. Chem. Technol. Biotechnol..

[48] Khan A., Saxena K.K. (2022). A Review on Enhancement of Mechanical Properties of Fiber Reinforcement Polymer Composite under Different Loading Rates. Mater. Today Proc..

[49] Kordi M., Farrokhi N., Pech-Canul M.I., Ahmadikhah A. (2023). Rice Husk at a Glance: From Agro-Industrial to Modern Applications. Rice Sci..

[50] Jabu M.A., Alugongo A.A., Nkomo N.Z. (2024). Application of Natural Fibre Composites in Interior Panels in the Automotive Industry: A Review. Int. J. Eng. Trends Technol..

[51] Mohammed L., Ansari M.N., Pua G., Jawaid M., Islam M.S. (2015). A Review on Natural Fiber Reinforced Polymer Composite and Its Applications. Int. J. Polym. Sci..

[52] Nikodinovic-Runic J., Guzik M., Kenny S.T., Babu R., Werker A., Connor K.E. (2013). Carbon-Rich Wastes as Feedstocks for Biodegradable Polymer (Polyhydroxyalkanoate) Production Using Bacteria. Adv. Appl. Microbiol..

[53] Coats E.R., Loge F.J., Wolcott M.P., Englund K., McDonald A.G. (2007). Synthesis of Polyhydroxyalkanoates in Municipal Wastewater Treatment. Water Environ. Res..

[54] Jiang G., Hill D.J., Kowalczuk M., Johnston B., Adamus G., Irorere V., Radecka I. (2016). Carbon Sources for Polyhydroxyalkanoates and an Integrated Biorefinery. Int. J. Mol. Sci..

[55] Chirdon W.M. (2015). Utilization of Biorefinery Waste Proteins as Feed, Glues, Composites, and Other Co-Products. Algal Biorefineries: Volume 2: Products and Refinery Design.

[56] Brebu M. (2020). Environmental Degradation of Plastic Composites with Natural Fillers—A Review. Polymers.

[57] La Mantia F.P., Morreale M. (2011). Green Composites: A Brief Review. Compos. Part A: Appl. Sci. Manuf..

[58] Dina I.A., Gimba C.E., Hamza A., Ekwumemgbo P. (2023). The Study of using Natural Fillers on the Biodegradation Properties of Virgin/Waste low Density Polyethylene and Virgin/Waste High Density Polyethylene Composites. J. Chem. Soc. Niger..

[59] Kaniuk Ł., Stachewicz U. (2021). Development and Advantages of Biodegradable PHA Polymers Based on Electrospun PHBV Fibers for Tissue Engineering and Other Biomedical Applications. ACS Biomater. Sci. Eng..

[60] Mohapatra S., Vishwakarma K., Joshi N.C., Maity S., Kumar R., Ramchander M., Pattnaik S., Samantaray D.P. (2021). A Review on PHAs: The Future Biopolymer. Environmental and Agricultural Microbiology, Applications for Sustainability.

[61] Saber D., Abdelnaby A.H. (2022). Recent Developments in Natural Fiber as Reinforcement in Polymeric Composites: A Review. Asian J. Appl. Sci. Technol. (AJAST).

[62] Elfaleh I., Abbassi F., Habibi M., Ahmad F., Guedri M., Nasri M., Garnier C. (2023). A Comprehensive Review of Natural Fibers and Their Composites: An Eco-Friendly Alternative to Conventional Materials. Results Eng..

[63] Koohestani B., Darban A.K., Mokhtari P., Yilmaz E., Darezereshki E. (2019). Comparison of Different Natural Fiber Treatments: A Literature Review. Int. J. Environ. Sci. Technol..

[64] Kolya H., Kang C.W. (2023). Next-Generation Water Treatment: Exploring the Potential of Biopolymer-Based Nanocomposites in Adsorption and Membrane Filtration. Polymers.

[65] Wang D.-C., Lei S.-N., Zhong S., Xiao X., Guo Q.-H. (2023). Cellulose-Based Conductive Materials for Energy and Sensing Applications. Polymers.

[66] Eissenberger K., Ballesteros A., De Bisschop R., Bugnicourt E., Cinelli P., Defoin M., Demeyer E., Fürtauer S., Gioia C., Gómez L. (2023). Approaches in Sustainable, Biobased Multilayer Packaging Solutions. Polymers.

[67] Musioł M., Janeczek H., Jurczyk S., Kwiecień I., Sobota M., Marcinkowski A., Rydz J. (2015). (Bio) Degradation Studies of Degradable Polymer Composites with Jute in Different Environments. Fibers Polym..

[68] Anwajler B., Zdybel E., Tomaszewska-Ciosk E. (2023). Innovative Polymer Composites with Natural Fillers Produced by Additive Manufacturing (3D Printing)—A Literature Review. Polymers.

[69] Jurczyk S., Musioł M., Sobota M., Klim M., Hercog A., Kurcok P., Janeczek H., Rydz J. (2019). (Bio) Degradable Polymeric Materials for Sustainable Future—Part 2: Degradation Studies of P (3HB-Co-4HB)/Cork Composites in Different Environments. Polymers.

[70] Musioł M., Jurczyk S., Sobota M., Klim M., Sikorska W., Zięba M., Janeczek H., Rydz J., Kurcok P., Johnston B. (2020). (Bio) Degradable Polymeric Materials for Sustainable Future—Part 3: Degradation Studies of the PHA/Wood Flour-Based Composites and Preliminary Tests of Antimicrobial Activity. Materials.

[71] Kamarudin S.H., Mohd Basri M.S., Rayung M., Abu F., Ahmad S., Norizan M.N., Osman S., Sarifuddin N., Desa M.S.Z.M., Abdullah U.H. (2022). A Review on Natural Fiber Reinforced Polymer Composites (NFRPC) for Sustainable Industrial Applications. Polymers.

[72] Markevičiūtė Z., Varžinskas V. (2022). Smart Material Choice: The Importance of Circular Design Strategy Applications for Bio-Based Food Packaging Preproduction and End-of-Life Life Cycle Stages. Sustainability.

[73] Jan-Georg R., Langer R., Giovanni T. (2022). Bioplastics for a Circular Economy. Nat. Rev. Mater..

[74] Phiri R., Rangappa S.M., Siengchin S., Oladijo O.P., Dhakal H.N. (2023). Development of Sustainable Biopolymer-Based Composites for Lightweight Applications from Agricultural Waste Biomass: A Review. Adv. Ind. Eng. Polym. Res..

[75] Naser A.Z., Deiab I., Darras B.M. (2021). Poly (Lactic Acid)(PLA) and Polyhydroxyalkanoates (PHAs), Green Alternatives to Petroleum-Based Plastics: A Review. RSC Adv..

[76] Melendez-Rodriguez B., Torres-Giner S., Aldureid A., Cabedo L., Lagaron J.M. (2019). Reactive Melt Mixing of Poly (3-Hydroxybutyrate)/Rice Husk Flour Composites with Purified Biosustainably Produced Poly (3-Hydroxybutyrate-Co-3-Hydroxyvalerate). Materials.

[77] Zhang K., Misra M., Mohanty A.K. (2014). Toughened Sustainable Green Composites from Poly (3-Hydroxybutyrate-Co-3-Hydroxyvalerate) Based Ternary Blends and Miscanthus Biofiber. ACS Sustain. Chem. Eng..

[78] Oliver-Ortega H., Julian F., Espinach F.X., Tarrés Q., Ardanuy M., Mutjé P. (2019). Research on the Use of Lignocellulosic Fibers Reinforced Bio-Polyamide 11 with Composites for Automotive Parts: Car Door Handle Case Study. J. Clean. Prod..

[79] Berzin F., Vergnes B. (2021). Thermoplastic Natural Fiber Based Composites. Fiber Reinforced Composites.

[80] Halley P.J., Dorgan J.R. (2011). Next-Generation Biopolymers: Advanced Functionality and Improved Sustainability. MRS Bull..

[81] Khamkong T., Penkhrue W., Lumyong S. (2022). Optimization of Production of Polyhydroxyalkanoates (PHAs) from Newly Isolated Ensifer Sp. Strain HD34 by Response Surface Methodology. Processes.

[82] Suhaily S.S., Khalil H.A., Nadirah W.W., Jawaid M. (2013). Bamboo Based Biocomposites Material, Design and Applications. Materials Science-Advanced Topics.

[83] Lange J.-P. (2021). Managing Plastic Waste—Sorting, Recycling, Disposal, and Product Redesign. ACS Sustain. Chem. Eng..

[84] Mishra T., Mandal P., Rout A.K., Sahoo D. (2022). A State-of-the-Art Review on Potential Applications of Natural Fiber-Reinforced Polymer Composite Filled with Inorganic Nanoparticle. Compos. Part C Open Access.

[85] Marciniak P., Możejko-Ciesielska J. (2021). What Is New in the Field of Industrial Wastes Conversion into Polyhydroxyalkanoates by Bacteria?. Polymers.

[86] Mohanty A.K., Misra M., Hinrichsen G.I. (2000). Biofibres, Biodegradable Polymers and Biocomposites: An Overview. Macromol. Mater. Eng..

[87] Wang C., Yu J., Lu Y., Hua D., Wang X., Zou X. (2021). Biodegradable Microplastics (BMPs): A New Cause for Concern?. Environ. Sci. Pollut. Res..

[88] Malafeev K.V., Apicella A., Incarnato L., Scarfato P. (2023). Understanding the Impact of Biodegradable Microplastics on Living Organisms Entering the Food Chain: A Review. Polymers.

